# Mirtazapine-induced neutropenic sepsis in an older person: a case report

**DOI:** 10.1186/s13256-023-03881-6

**Published:** 2023-04-14

**Authors:** Alice Maidwell-Smith, Charlotte Kirk

**Affiliations:** 1 University Hospitals Sussex NHS Foundation Trust, Worthing Hospital, Lyndhurst Rd, Worthing, West Sussex BN11 2DH UK; 2 Hampshire Hospitals NHS Foundation Trust, Royal Hampshire County Hospital, Romsey Rd, Winchester, Hampshire SO22 5DG UK

**Keywords:** Mirtazapine, Antidepressant, Neutropenia, Older person, Geriatrics, Case report

## Abstract

**Background:**

Mirtazapine is a frequently prescribed psychotropic drug for depression in older age. It is considered safe and has a side-effect profile uniquely favorable to an older person affected by reduced appetite, difficulty maintaining body weight, or insomnia. However, it is largely unknown that mirtazapine can cause a dangerous decline in neutrophil count.

**Case presentation:**

We present a case of mirtazapine-induced severe neutropenia in a 91-year-old white British woman requiring drug withdrawal and granulocyte-colony stimulating factor administration.

**Conclusion:**

This case is of significance because mirtazapine is regarded as a safe, and often preferable, antidepressant in older age. However, this case demonstrates a rare, life-threatening side effect of mirtazapine and calls for greater pharmacovigilance when prescribing it. There is no previous report of mirtazapine-induced neutropenia requiring drug withdrawal and granulocyte-colony stimulating factor administration in an older person.

## Background

Antidepressant drugs are used in the treatment of depression and their efficacy in an older population is well substantiated. However, age-related pharmacodynamic and pharmacokinetic changes mean side-effect profiles must be carefully considered. Furthermore, drug–drug interactions, comorbidities, and frailty must all be taken into account.

Selective serotonin reuptake inhibitors (SSRIs) are regarded as first line in the pharmacological treatment of depression [1]. However, the anticholinergic burden and the risks of hyponatremia, QT interval prolongation, and gastrointestinal bleeding can be troublesome in the older person. Consequently, mirtazapine, an atypical antidepressant, is frequently used as an alternative [1]. The common side effects of mirtazapine, including increased appetite and sedation, may also be advantageous in some older individuals. However, there is little clinical awareness of the risk of neutropenia when prescribing mirtazapine. We report a case of mirtazapine-induced severe neutropenic sepsis in an older person.

## Case presentation

A 91-year-old white British woman attended hospital via the primary care out-of-hours service with a fall and a “long-lie” of 11 hours. She had a background of atrial fibrillation, dual-chamber pacemaker, asthma, chronic kidney disease stage four, and depression. Her regular medications included apixaban, omeprazole, mirtazapine, and formoterol and salbutamol inhalers. There was no history of previous blood dyscrasias.

**Fig. 1:**
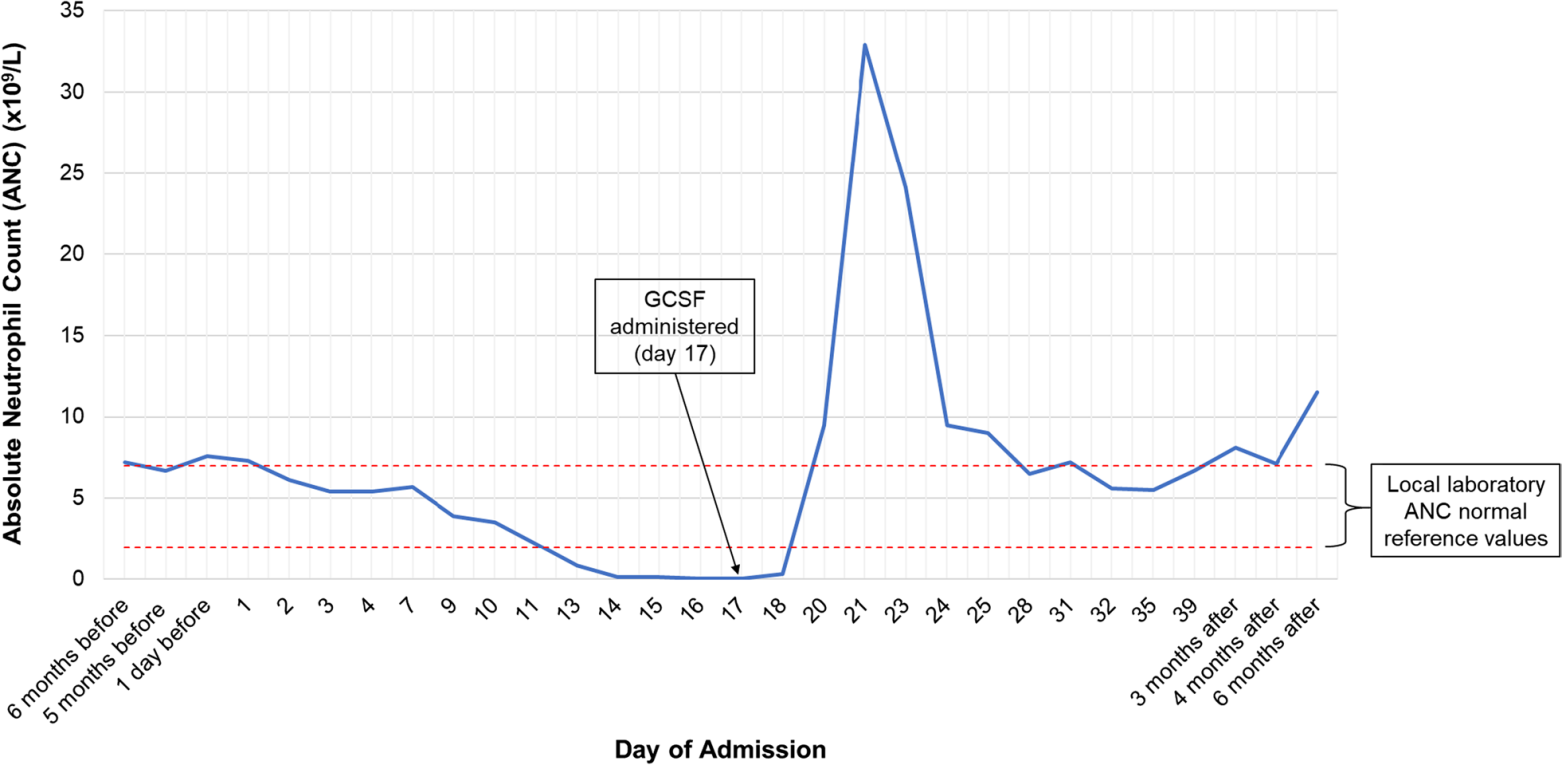
Absolute neutrophil count in relation to day of admission

Clinical examination at first presentation was unremarkable. Blood tests demonstrated raised inflammatory markers and acute kidney injury, requiring admission and intravenous fluids. During her admission, an asymptomatic, unexplained decline in absolute neutrophil count (ANC) was observed, resulting in undetectable neutrophils by day 17 (Fig. 1). She later developed the clinical signs of neutropenic sepsis and required intravenous antibiotics and granulocyte-colony stimulating factor (GCSF), which prolonged her hospital stay.

Basic hematological investigation such as vitamin B12, folate, and a blood film failed to identify a cause of neutropenia. However, review of medications revealed mirtazapine 15 mg nightly was commenced 3 weeks prior to admission. In the absence of other etiology, a diagnosis of mirtazapine-induced neutropenia was made. Mirtazapine was subsequently discontinued and her ANC improved from day 21 (Fig. 1). She went on to make a good clinical recovery and was discharged. Further blood monitoring demonstrated her ANC remained within normal parameters up to 6 months post-discharge (Fig. 1).

## Discussion

While mirtazapine is commonly prescribed in older adults, neutropenia (ANC < 1.8 × 10^9^/L) is a rare but dangerous complication. Mirtazapine-induced neutropenia was first demonstrated during pre-marketing trials where 11 per 10,000 patients developed severe neutropenia (ANC < 0.5 × 10^9^/L) between 9 and 61 days after drug initiation [2]. However, the incidence of neutropenia of lesser severities was not disclosed. The variation in time to onset of mirtazapine-induced neutropenia suggests more than one mechanism of action, although the pharmacodynamics have yet to be established.

More recently, mirtazapine-induced neutropenia has been reported twice in patients > 70 years of age [3, 4]. In these reports, patients’ neutrophil count was affected between 10 days and 8 months of mirtazapine exposure and improved rapidly with its discontinuation [3, 4]. However, our report uniquely describes the development of an undetectable neutrophil count requiring GCSF administration. To our knowledge, this is the first report of mirtazapine-induced neutropenia requiring drug withdrawal and GCSF in an older person. Furthermore, we have demonstrated normal ANC levels 6 months following discontinuation of mirtazapine, which is a particular strength of this case report.

Notably, mirtazapine use alongside other possible culprit medications has been recognized in a fatal case of neutropenic sepsis [5]. Therefore, it is important that clinicians are aware of this serious side effect to aid early recognition and management. Patients prescribed mirtazapine should be advised to report any signs of infection to their healthcare professional, who should perform a blood count and discontinue the drug immediately if a blood dyscrasia is suspected [6]. The manufacturers recommend mirtazapine be discontinued if there are clinical “signs of infection, with a low white blood cell count” [2]. This is particularly pertinent in older adults with reduced physiological reserve. However, in older adults it would be pragmatic to discontinue mirtazapine with any acute fall in ANC before infection develops. As suggested by this report and the surrounding literature, withdrawing mirtazapine would likely lead to resolution of neutropenia.

## Conclusion

This case illustrates how severe neutropenic sepsis developed less than a month after commencing mirtazapine in an older person, requiring drug withdrawal and GCSF administration. Furthermore, we demonstrate complete resolution of neutropenia following discontinuation of mirtazapine. This report highlights a rare but life-threatening side effect of mirtazapine, a commonly prescribed psychotropic for depression in older adults, and calls for greater pharmacovigilance when prescribing it.

This case report does not provide sufficient evidence to justify hematological monitoring of all patients starting mirtazapine. However, we speculate future investigation into monitoring for blood dyscrasias would be warranted should further reports of mirtazapine-induced neutropenia emerge.

## Data Availability

Not applicable.

## Abbreviations

ANC: Absolute neutrophil count
GCSF: Granulocyte-colony stimulating factor
SSRIs: Selective serotonin reuptake inhibitors
